# Intravitreal Injection-Induced Migraine Headaches

**DOI:** 10.7759/cureus.561

**Published:** 2016-04-06

**Authors:** Valerie C Lerebours, Thanh-Giao Nguyen, Vimal Sarup, Fabian Rossi, Saad Shaikh

**Affiliations:** 1 Ophthalmology, Howard University Hospital; 2 Ophthalmology, Orlando VA Medical Center; 3 Ophthalmology, UCF College of Medicine; 4 Neurology, Orlando VA Medical Center; 5 Ophthalmology, USF College of Medicine; 6 Ophthalmology, Howard University College of Medicine; 7 Ophthalmology, University of Texas Medical Branch at Galveston; 8 Ophthalmology, Florida State University College of Medicine

**Keywords:** cephalgia, intravitreal injection, migraine, neuralgia, headache, retrobulbar injection

## Abstract

A case of migraine headache triggered by intravitreal injection, and aborted by retrobulbar injection, is reported. To date, migraine and related cephalgia have not been reported after intravitreal injection. Ophthalmologists and neurologists should be aware of this potential sequela of a very common procedure.

## Introduction

Ocular pain is a common finding after an ophthalmic procedure. It can be induced by inflammation, ocular surface irritation, an increase in intraocular pressure, amongst other etiologies. Ocular pain and headaches may also be associated with migraine pathology. We present a case of a known migraineur in whom episodes were triggered by intravitreal injection. To our knowledge, this is the first such association in medical literature.

## Case presentation

A 57-year-old male was seen in the eye clinic for an intravitreal injection of ranibizumab for macular edema secondary to a nonischemic central retinal vein occlusion. The patient’s medical history was significant for Behcet’s disease, diabetes mellitus, chronic renal disease, and hemophilia. He also suffered from episodic migraines, always right-sided, occipital and temporal in distribution, with associated photophobia and nausea. He did not report other ocular or facial autonomic symptoms or auras. The patient received an intravitreal injection in the right eye without complications. Topical anesthesia with 4% lidocaine was administered to achieve anesthesia before injection, which was administered pain free. Four hours post injection, he was awakened by severe right eye pain accompanied by headache that resembled his migraines. He went to the emergency room where he received minimal relief with opioid medications. The patient was seen in the eye clinic the next day with reported 10/10 lancinating right eye pain, extreme photophobia, nausea, and ipsilateral headache. Examination revealed no periorbital injection or inflammation, no obvious anterior chamber reaction, no evidence of corneal defects, and outside of a small hemorrhage in the area of injection, an otherwise quiet conjunctiva and sclera. A normal pupillary response was present without afferent pupillary defect. The intraocular pressure was within normal limits. The patient's pain and photophobia made a thorough eye examination impossible, and for this reason, after a cursory non-dilated fundus exam was confirmed to be normal, a retrobulbar injection of 2% lidocaine was performed. His eye pain and headache resolved completely within minutes of administration, and the patient was able to cooperate with the remainder of his eye exam. Dilated funduscopic examination demonstrated a clear vitreous, and a well perfused optic nerve. Macular edema, retinal vascular tortuosity, and retinal hemorrhages were present consistent with central retinal vein occlusion. The patient remained pain free when he was contacted the following morning. This was the patient’s seventh intravitreal injection. He reported a similar episode of eye and head pain on the first injection which responded well to intravenous infusion of Dilaudid. The intervening five injections did not result in pain. The patient was referred back to his neurologist for additional prophylactic and abortive medical management of his migraines. He was placed on a regimen of topiramate and has received six additional intravitreal injections without onset of headache. 

## Discussion

The International Classification of Headache Disorder, Third Edition, beta version (ICHD 3) established the diagnosis criteria of migraine without aura as at least five attacks of headache lasting 4–72 hours (untreated or unsuccessfully treated), with two of the following four characteristics: unilateral location, pulsating quality, moderate to severe pain intensity, aggravated with physical activity, and at least one of the following: nausea and vomiting or photophobia and phonophobia [1]. The pathophysiology of migraines is not completely understood but trigeminal activity appears to play a significant role [2]. In addition, the classification of migraines is difficult as they share features with other trigeminal cephalgias. Trauma is a known trigger of such headaches, as is ocular surgery [3-4]. Intravitreal injection is a common ophthalmic procedure. Our patient received an intravitreal injection with a 32-gauge needle in the superotemporal portion of the globe which we believe triggered at least two migraine headache episodes. He presented with symptoms of nausea, photophobia, and pain that are commonly seen when postoperatve complications such as corneal abrasion, inflammation and/or intraocular pressure elevation are present. None of these findings were noted on his examination and his pain response was well out of proprtion to that observed with intravitreal injections. The injection may have irritated his ciliary nerves triggering a trigeminal mediated migraine or related cephalgia [4]. Our patient’s headache responded to anesthetic administered in the distribution of the trigeminal nerve which has previously, although not via retrobulbar route, been reported as an abortive intervention for migraines and other trigeminal headaches [2]. Migraine triggers are often inconsistent and unpredictable. In patients with a migraine history not every intravitreal injection may induce a pain episode, as was the case here.

## Conclusions

Physicians should be aware that intravitreal injections may induce headaches in migraine and related trigeminal headache patients and they should be consented accordingly. Additional abortive medications and measures may be necessary and close management with a headache specialist is recommended. In addition, although we do not recommend it as routine practice, retrobulbar anesthetic injection appears to have an abortive role in the management of trigeminal headache.
